# COVID-19 Testing: A Qualitative Study Exploring Enablers and Barriers in the Greater Accra Region, Ghana

**DOI:** 10.3389/fpubh.2022.908410

**Published:** 2022-07-12

**Authors:** Solip Ha, Sonam Yangchen, Abraham Assan

**Affiliations:** ^1^Seoul National University Graduate School of Public Health, Seoul, South Korea; ^2^Institute of Health Partners, Thimphu, Bhutan; ^3^Global Policy and Advocacy Network (GLOOPLAN), Accra, Ghana

**Keywords:** COVID-19 testing, barriers, facilitators, qualitative study approach, pandemic preparedness, Ghana

## Abstract

**Introduction:**

The COVID-19 pandemic is a global crisis and has reaffirmed that extensive testing along with effective tracing is still crucial to slowing transmission of the COVID-19 virus despite the rolling out of vaccines. This study explored enablers and barriers to COVID-19 testing in the Greater Accra region of Ghana. We envision lessons learned from this study could serve as a road map to strengthen the current response to COVID-19 and preparedness for future outbreaks, particularly in low- and middle-income countries.

**Methods:**

A qualitative design was undertaken to explore the phenomenon. Data collection methods included in-depth interviews with key informants with a purposively selected sample of 20 participants. Interviews were conducted using flexible semi-structured interview guides. Depending on the participant's position and involvement in COVID-19 testing, the guides were modified, and key elements were drawn from a tailored version of the WHO Health System Framework, incorporating the Essential Public Health Functions (EPHF). The interview findings were augmented by reviewing published literature.

**Results:**

Better health governance through political leadership, community participation, multisectoral collaboration, effective resource management, and information systems played a crucial role in catalyzing COVID-19 testing. The primary barriers to testing were mainly COVID-19 infodemic, inadequacy of material resources to meet growing health needs, and a lack of opportunities to have equal and easy access to testing services. Furthermore, although human resources were adequate, they were unevenly distributed across settings.

**Conclusion:**

Despite rolling out vaccines against COVID-19, testing remains an important measure to control the virus. To effectively be prepared for extensive COVID-19 testing and respond to future outbreaks, the following are recommended: there should be improved political commitments, coordination, and communication with diverse actors to ensure even distribution of all resources across the country; empowerment of community members should be encouraged to develop community-oriented pandemic preparedness and management of COVID-19 infodemic; investment in strengthening capacity of Good Manufacturing Practice (GMP); incorporation of health policy and systems research (HPSR) into the post-COVID-19 pandemic recovery process and future pandemic preparedness.

## Introduction

The Coronavirus Disease 2019 (COVID-19) pandemic has dominated headlines worldwide since first reported in 2019, and the most recent emergence of a new COVID-19 virus variant (omicron) has exacerbated the global health crisis. According to the World Health Organization (WHO), there have been 396 million confirmed cases of COVID-19, including 5.7 million deaths, and 10 billion vaccine doses have been administered as of 8 February 2022 (1). Although vaccines are rolled out in many countries to help reduce the risk of contracting the virus and infecting others, they continue to experience second and third waves, and, thus, extensive testing strategies still remain essential to slowing transmission (2). Besides, COVID-19 testing could be a potentially less disruptive management strategy, particularly where vaccine access is limited (2). Increasing testing capacity enables resource-poor countries experiencing slow rollout of vaccines due to poor supply chains to break disease transmission and reform the architecture for future pandemic preparedness efficiently (3).

Several countries adopted strict measures at the beginning of the pandemic to contain the spread of the virus. Ghana, a lower-middle-income country in West Africa with a population of about 30 million, recorded its first case on 12 March 2020 (4). But even before that, the country had already implemented early preventive strategies, such as the 3T approach (tracing, testing, and treating) in response to the pandemic (4). Efforts were geared toward the adoption of multisectoral actions with wide control measures to prevent, detect, and contain the disease. The private sector, civil societies, and faith-based organizations all played a key role in this regard. For example, through the private sector, the government is putting up a 100-bed hospital to be completed within 6 weeks for the isolation and treatment of patients with COVID-19 (5, 6). Moreover, Ghana's longstanding efforts to strengthen Primary Health Care (PHC) through the Community-Based Health Planning and Services (CHPS) program, and its National Health Insurance Scheme (NHIS) (7) altogether contributed to the success of extensive testing of which the WHO is even studying some of the techniques (8).

Ghanah is one of the highest testing rates in sub-Saharan Africa (4, 9). Comparing tests conducted as per the million population in sub-Saharan Africa, Ghana has made huge progress in testing a large number of the population (4, 9). Its daily tests per million are 100, which is the second-largest test performed as of 2 March 2022 (4, 6, 9). This might be the result of the president's early policy decision to enhance the capacity to test and expand the numbers of testing, treatment, and isolation centers (10). Furthermore, Ghana used the overarching contact tracing approach, which has now become a model for managing COVID-19 (10), and the approach worked well within the Ghanaian context. However, it has also been constrained by the issue of resources, infrastructure, and the COVID-19 infodemic.

Despite the importance of scaling up COVID-19 testing as cost-effective prevention, isolation, and detective measure, there is very little literature, including some articles, commentaries, and opinions exploring the effect of factors influencing the testing in the context. Previous research and its findings on COVID-19 testing have generally prioritized biomedical and clinical aspects of the testing. For instance, Lopes-Júnior et al. conducted a systematic review to synthesize and critically evaluate the scientific evidence on the influence of the testing capacity to control COVID-19. This study found a reproducible strategy to query the scientific literature on the effectiveness of mass testing for the control of COVID-19 in a clinical context (11). Other qualitative research on perspectives on COVID-19 testing policies and practices highlighted tensions between communications and implementation of testing developments and uncertainties about the responsibility for testing and its implications focusing on the different health actors (12–14).

These studies have concentrated on assessing the effectiveness of COVID-19 testing in biomedical and clinical aspects and identifying challenges primarily focused on health professionals and practitioners for mass testing, but there has not previously been an attempt to identify influencing factors to scaling up COVID-19 testing, considering all different aspects. Overall, current research on COVID-19 testing provides little evidence on how to expand testing to contain the virus, particularly in resource-poor settings. Understanding the reasons behind COVID-19 testing challenges may inform better strategies to address them. This qualitative study examines facilitators and barriers to scaling up COVID-19 testing in Ghana, focusing on the capital city region, Greater Accra, by investigating the country's response. The different narrative perspectives enabled this study to acknowledge diverse experiences from interview participants to identify multifaceted, complex, but not observable factors. This study aims to fill a knowledge gap in understanding key drivers to increase COVID-19 testing and identify what challenges have influenced Ghana's multi-leveled and cross-sectoral responses to the pandemic. The lessons from Ghana's case can benefit other resource-poor settings to contain emerging pandemics and strategize future prevention of infectious diseases.

## Methods

### Study Design, Sampling, and Data Collection

A qualitative design was undertaken to explore the barriers and facilitators to scaling up COVID-19 testing implementation in Greater Accra, Ghana. It is the capital city of the country, with a population of about 2.6 million (15, 16). A purposive sampling approach was used to recruit 20 key informants for interviews from September to October 2021. The participants had provided written consent before being interviewed. All interviews were conducted virtually through a Zoom platform by authors SH and SY. The study participants comprised policymakers, implementers, frontline workers, and community members. The policymaker and implementer groups were selected, considering their positions and influence in decision-making, implementation, and evaluation of COVID-19 testing in Ghana (Table 1). The interviews lasted between 40 and 60 min and were audio-recorded.

**Table 1:** Characteristics of participants.

**Key Participants**	**Characteristic**	**Total**
Policymakers	Senior officials at Policy, Planning, Monitoring and Evaluation unit at the Ministry of Health (2)	2
Implementers	Laboratory managers from medical research institutions (6)	15
	Laboratory scientists from COVID-19 testing centers (4)	
	Researchers from medical research institutions and Universities (5)	
Frontline workers	Nurses from hospitals (2)	2
Community members	Community leader in Greater Accra (1)	1
Total		20

The interviews were conducted using flexible semi-structured interview guides. Depending on the participant's position and involvement in COVID-19 testing, the guides were modified, and key elements were drawn from the WHO Health System Framework, incorporating the Essential Public Health Functions (EPHF) (17).

The sample size reached data saturation, where no more new information was discovered in data analysis. All researchers agreed on four general principles and concepts regarding data saturation: no new data, no new themes, no new coding, and the ability to replicate the study (18). The researchers were confident that the collected data were sufficient, consistent, and qualified enough to meet the objectives. All the interviews were conducted in English. In addition, a desk review of the existing literature about Ghana's national health policies and systems, governance, and national COVID-19 preparedness and response plan was conducted to triangulate interview data and understand the context to conceptualize key themes from the collected data (Table 2).

**Table 2:** Reviewed documents and data analyzed.

**Documents selected**	**Data analyzed**
Policy responses to fight COVID-19; the case of Ghana (4)	Ghana's national health policies and systems, governance
Ghana's COVID-19 response: the Black Star can do even better (5)	Health systems and governance for COVID-19 response
Containing the impact of COVID-19: Review of Ghana's response approach (6)	National COVID-19 preparedness and response plan and Ghana's health systems
How well is Ghana with one of the best testing capacities in Africa-responding to COVID-19? (8)	National COVID-19 preparedness and response plan and testing capacities
‘Test and trace’ has worked for us, Ghana's President says (10)	Importance of 3Ts (test, tracing and treating) in Ghana
Saturation in qualitative research: exploring its conceptualization and operationalization (14)	Conceptualization of key themes from qualitatively collected data
How digital technology helped support Ghana's COVID response (16)	Health systems and surveillance using digital technology
Ghana receives critical COVID-19 medical supplies (18)	Health supplies and resource distribution

### Data Processing and Analysis

The interviews were audio-recorded. To accurately capture the detail of fully recorded data, SH and SY independently transcribed all the audio recordings verbatim. We combined both inductive and deductive approaches to coding. We employed the framework by following these steps: (a) familiarization; (b) identifying initial codes; (c) indexing; (d) generating main, sub-themes, and codes; and (e) defining and interpreting themes. In addition, we remained open to accommodate other themes. Coding was conducted manually, and a codebook was developed to identify and document all existing thematic categories. The selected core categorical themes were analyzed and compared with existing literature to ensure our findings' reliability, validity, and comprehensiveness (18). It is important to note that the thematic categories were formed based on previously conducted qualitative research on COVID-19 and acute respiratory illness (12–14).

## Results

Characteristics of the participants are shown in Table 1. The participants comprised policymakers, implementers, frontline workers, and community members. Most of the participants were implementers (75%) working in COVID-19 testing facilities and medical research institutions.

A total of 6 themes related to facilitators and barriers to scaling up COVID-19 testing, 12 sub-themes, and 26 codes were derived from the analysis and coding process (Table 3). The results of the study are structured and presented in sections according to the main thematic areas and supported with quotes.

**Table 3:** Main, sub-themes, and codes derived from the coding process.

	**Themes**	**Sub-themes**	**Codes**
**Facilitators**	Health governance and leadership for navigating COVID-19	Political leadership	• High political commitment to increase testing • High involvement of the President of Ghana to tackle the pandemic by increasing testing • High governmental effort for understanding the implementation of health regulations
		Community leadership	• Community leaders and organizations' commitments to enhancing adherence to COVID-19 protocols and testing • Religious leader's high commitment to educating community members to adhere to health regulations and get tested
		Multisectoral partnerships and collaboration	• High involvement of diverse entities from different sectors for financial support and distribution of health supplies
	Resource management	Human resources	• Training programs to strengthen health workers' capacity and ensure an adequate number of health workers • Availability of incentive packages for health workers • Positive attitude and behaviors of health workers toward work
		Financial and material resources	• High financial commitments on the part of the government to ensure adequate financial and material resources • Availability of testing centers
	Information system	Surveillance systems	• Existence and use of a centralized data reporting system • Well-managed procurement and surveillance system
		Health communications	• Successful advocacy campaigns to ensure adherence to health protocols • Provision of regular updates on COVID-19 by the governmental authorities and health institutions
**Barriers**	Resources	Inadequate human resources to respond to growing needs	• Inadequate human resources in COVID-19 testing centers leading to a heavy workload and its related consequences such as anxiety
		Financial and Material resources barriers to testing	• Low internet connection to manage data • Poor transportation infrastructure to deliver test samples • High price of COVID-19 testing • High dependency on reagents and consumables from other countries • Occasional shortage of reagents and testing kits
	COVID-19 infodemic	Socio-cultural perspectives and practices	• Strong reliance on negative experiences of people about COVID-19 testing to refuse testing
		Misconceptions about COVID-19 and testing	• Misinformation about COVID-19 • Low level of knowledge about COVID-19 leading to misconceptions, especially among rural residents and women
		Fear of stigmatization for testing positive	• Fear, anxiety and isolation associated with testing positive in the community
	Service delivery		• Lack of opportunities for all community members to have equal and easy access to testing services

## Facilitators of COVID-19 Testing

### Health Governance and Leadership for Navigating COVID-19

#### Political Leadership

The study revealed how effective leadership from the Government of Ghana positively influenced efforts to foster COVID-19 response strategy. Many participants confirmed the important role of political leadership in enhancing the general understanding of the Ghanaian population about the COVID-19 pandemic and response mechanisms, such as adhering to health regulations and getting tested. They reported that the president's guidance strongly influenced a sense of collective responsibility and encouraged people to adhere to response strategies, particularly on being tested against the virus. More importantly, the participants emphasized that leadership and coordination between the government and the local authorities fostered a sense of collective responsibility and timely responses to the threats posed by the pandemic.

“*We received lots of hope from the government and the president. Our president was really keen on tackling the pandemic, and, with a lot of support from other governmental entities, such as the Ghana Health Service and the National Commission for Civic Education (NCCE), we could respond to the COVID-19 pandemic timely. We were highly encouraged to get tested by the president, and his leadership uplifted the motivation of getting tested*.”- A researcher from medical research institute 1.

#### Community Leadership

The majority of the participants agreed that strong community leadership played an important role in distributing personal protective equipment (PPE), sensitizing health protocols and encouraging vulnerable groups to actively engage in the community to fight against COVID-19. According to our findings, community leaders and members played a critical role in successfully managing the pandemic through close collaboration with the Ghana government, health institutions, non-governmental organizations (NGOs), and other faith-based organizations. Furthermore, community leaders, including religious leaders, helped provide public health education for members of their community using their local languages. This enabled community members, especially the illiterate, to understand the COVID-19 burden and the consequences of not adhering to health protocols.

“*Many religious leaders from the churches are currently educating people to follow all COVID-19 protocols, such as wearing masks, washing hands, and getting tested on the virus. We are doing our best to educate our members to follow the protocols and get tested timely. We also help them to disseminate identified information related to availing testing”-* Community Leader 1.

#### Multisectoral Partnerships and Collaboration

Our findings revealed a high level of multisectoral partnership and engagements of diverse stakeholders, such as intergovernmental organizations, religious institutions, civil society, and the private sectors. The study participants recounted how engagement with the private sectors for financial and material support enhanced COVID-19 testing capabilities, including setting up major testing centers across the country and ensuring testing kits and supplies. For example, Nyaho Medical Center and Frontiers Health Care Services are major private testing centers in the country. The development partners also assisted with the provision of laboratory equipment and the logistics for testing.

“*… we have been supported by various private companies, NGOs, and ministries. The private companies and some NGOs helped some vulnerable groups with health supplies and funds for COVID-19 testing. My institution was also given some financial and material support from them, such as reagents for COVID testing*.”—Laboratory Manager 1.

### Resource Management

#### Human Resources

The study participants indicated that there are sufficient health professionals, training programs, and platforms to enhance the capacity of the health workforce to meet up to tasks fully. Also, the interviews highlighted that there were several incentive packages for health workers, including insurance packages and tax relief (19). According to the opinions of some participants, this enabled them to exhibit a positive attitude at work. Also, the training improved the confidence level and interpersonal relationships between health professionals and their clients.

“*We have been provided extensive training and go through a series of training for 3 weeks or 1 month. We learn everything from whole processes of sample collecting to how to make patients calm down to make sure that they are comfortable when taking a nasal pharyngeal swab.” -* Laboratory Scientist 1.

#### Financial and Material Resources

Provision of financial resources is the key to ensuring adequate health supplies to foster a timely response to COVID-19. The participants mentioned the commitments of the Ministry of Health (MoH) to ensuring adequate financial and material resources in upscaling testing. They further emphasized that support from individual donors, international organizations, and NGOs enabled the government to achieve this goal.

“*We did not have enough testing centers and PPE at the beginning of the pandemic. But, now, we have enough facilities, adequate PPE, and other consumables supported by the Ghana government, international organizations, and other donors for COVID-19 testing. For example, the Ghana airport testing center has been recently established and offers COVID testing at the Kotoka International Airport.”—*Laboratory Manager 2

Furthermore, most of the participants emphasized that the availability of more testing centers, infectious disease centers, sustainable funding initiatives, such as the establishment of the COVID-19 National Trust Fund (CNTF), and funding programs for enterprises all contributed to successful COVID-19 responses in Ghana, including testing. A senior official at MoH mentioned that “*We established COVID-19 Alleviation and Revitalization of Enterprises Support (CARES) program to mitigate the impact of the pandemic on the livelihoods of Ghanaians and support businesses and workers, and introduced a package of economic stimulus measures called the Coronavirus Alleviation Programme (CAP) to formulate and implement the COVID-19 preparedness and response plan, tracing, testing, and treatment.”*

### Information System

#### Surveillance Systems

The participants revealed the establishment of a central procurement system, and a surveillance system was vital to COVID-19 preparedness and response, and provision of essential services. A senior officer at MoH said, “*We have a good surveillance system to rapidly detect, test, and manage cases to monitor the virus, and Ghana Health Service can see all available data at one glance using it. We also have a good, systemized procurement system through procurement agencies.”*

Furthermore, real-time surveillance of reported COVID-19 infections has been the key to the global pandemic response. Many tools, devices, and apps have supported surveillance in Ghana (20). The participants emphasized the role of the centralized data reporting system in Ghana called Surveillance Outbreak and Response Management and Analysis System (SORMAS). According to interviewees, the system enabled health professionals to identify defaulters and get them tested. They also pointed out that it helped monitor stock to avoid shortages of materials for testing, such as test kits and PPE.

“*The Ghana Health Service traces all positive cases based on collected data. It helped connect all labs across districts to access to it, also, helped detect, investigate, and control the virus in the long run*.”—Laboratory Manager 3

#### Health Communications

Public health communication plays an important role in protecting public health during pandemics. According to the interviews, the government of Ghana effectively provided information through various public media for the people, particularly by educating them on measures that need to be undertaken to contain the spread of the virus. The participants asserted that mass media campaigns on adherence to health protocols and regular updates on COVID-19 by the president and health authorities positively influenced community members to adhere to health directives. A researcher from the University pointed out, “*There are several TV and radio advertisements, and they are compelled to educate people. The advertisements ensured that concepts and materials on COVID-19 testing are seen as culturally relevant and well understood by people.”*

## Barriers to COVID-19 Testing

### Resources

#### Inadequacy of Human Resources (Testers) to Respond to Growing Needs

Interestingly, 55% of the participants agreed that the government's effective interventions in the health sector helped bridge the gap between demand and supply of human resources, while the same proportion of the participants (55%) complained that, despite the government's effort, there is still an issue of limited human resources, especially in COVID-19 testing centers. A laboratory manager recounted, “*We have only one person at the lab who runs the test. Despite our support, he ran samples until late. I also feel too exhausted and tired when testing many people. The human personnel is fewer. One of the reasons is that some people think it is too dangerous, and, maybe, they can use the same expertise to bring more changes in the science field, Ghana.”*

Some health professionals also revealed that they experience high stress and burnout due to a heavy workload during the prolonged pandemic. A nurse said, “*I think we have been demotivated for the past years. We are risking our lives during the pandemic, and, sometimes, it makes us miserable and stressful despite our passion for patients.”*

#### Financial and Material Resources Barrier to Testing

According to findings of the study, poor infrastructures, such as low internet connectivity and an inadequate transportation system, posed a challenge to effective delivery of collected blood samples, thereby delaying test results and data loss. Again, there was difficulty in transporting tools and equipment due to the poor roads.

“*The main issue is logistics and transport to deliver blood samples to national labs in Accra (the capital city and where testing labs are located). When we were supposed to leave to take samples outside from the community, the car wasn't ready. This was the challenge during the collection of samples and taking samples to Accra.”*—Laboratory Manager 4

The Ghana government received COVID-19 medical supplies from international organizations to be given to hospitals and clinics to scale up the COVID-19 response effort in 2021 (21, 22). However, the majority of the participants asserted that the cost of being tested was high for most people, thereby deterring people from being tested. A laboratory scientist mentioned, “*The government should do something about COVID-19 tests by reducing the price or making it free for the ordinary people because most people who do not have a prescription from doctors are reluctant to pay expensive testing fees and get tested.”*

Also, the participants indicated that the high dependency on imported reagents and consumables from other countries poses a challenge to an effective response to the growing threats, especially whenever such products become scarce in the donors countries. According to the participants, this contributed to the occasional shortages of material resources, such as reagents and testing kits. A researcher at the medical research institute reported, “*There is, currently, a shortage of health supplies for testing. This occasionally happens when more people want to get tested and know their status. So, securing enough testing kits and reagents is very important.”*

### COVID-19 Infodemic

#### Sociocultural Perspectives and Practices

This study revealed a salient aspect of how a sociocultural perspective of the members of the Ghanaian community influenced testing and general response to COVID-19. The participants expressed a dilemma as to how people are impacted by Ghanaian culture positively and negatively. The participants mentioned some positive cultural impacts on COVID-19, such as practice of hand hygiene (23). On the other hand, the majority of the study participants mentioned other negative sociocultural influences on people's behavior and beliefs toward adherence to COVID-19 protocols, for example, a high dependency on neighbors' negative personal experiences about COVID-19 testing rather than accurate information from the health experts. Thus, people would like to be tested based on shared experiences of their close friends and relatives. A laboratory scientist mentioned, “*Ghanaians are particular about maintaining relationships with family and friends. So, when the pandemic occurred, people shared their uncomfortable experience with nasal swabs for testing, and some of them have a fear of getting tested.”*

#### Misconceptions About COVID-19 and Testing

Almost all the participants pointed out misconceptions about COVID-19. They emphasized that some people hesitate to get tested because they consider COVID-19 as just common flu. Furthermore, some people believe in rumors that coronavirus does not exist. According to our findings, these factors influenced the decision of community members to be tested for COVID-19.

“*Some people believe in fake news and misinformation that the virus is not real, so vaccination and testing are unnecessary. Also, they think COVID-19 is just like flu, and big countries are using it for political purposes*”—A Community Leader 1.

“*I have a patient who strongly believes that the virus does not exist. So, I think educating them to adhere to health protocols using TV and radio is essential.”*—A Nurse 1

Furthermore, the participants narrated that most women in the community do not have the same level of knowledge or information about the virus as men and how to adhere to health protocols. A senior officer at MoH cited, for example, that the level of educated men is higher compared to women, “*Education will make a lot of difference in the heart of people concerning the COVID-19 and its right information. However, at the same time, I feel that illiteracy among women can be a barrier, especially in rural areas. Unfortunately, they (women) have fewer opportunities to be educated than men.”*

#### Fear of Stigmatization for Testing Positive

Some participants also shared their experiences with patients and community members. According to them, most people were afraid of being isolated and stigmatized by their members of the public, including their workplaces and community. The respondents highlighted the feeling of guilt and shame for people testing positive, which deters others from coming for testing. These findings support studies showing that healthcare workers, COVID-19 recovered patients, and suspected persons of COVID-19 have faced various forms of COVID-19-related stigma and discrimination, such as stereotyping, social exclusion, mockery, finger-pointing, and insults in Ghana (24, 25).

“*If you are diagnosed as COVID-19 positive, people will not even allow you to go to this particular neighborhood; for example, as a COVID-19 worker, people think I will also be infected by the virus one day. So they try not to be close to me.”—*Laboratory Scientist 2

### Service Delivery

Ghana's health care structure is regarded as well developed compared to other countries in sub-Saharan Africa (4). There are five levels of providers, including health posts, health centers and clinics, district hospitals, regional hospitals, and tertiary hospitals, to improve accountability to local population, efficiency in service provision, equity in access and resource distribution, and increased resource mobilization (26). However, this decentralized system and its functions have not been as effective as they could have been in the outskirts of Accra. A nurse working at a remote hospital from Greater Accra said, “*Like mining town, everybody says they do not have equal opportunities to access good healthcare services compared to the capital city, Accra. Full attention and support for the people in rural areas is really needed*.”

## Discussion

The findings from this study illuminate the facilitators and barriers to scaling up COVID-19 testing in Ghana. A combination of leadership from the community level to the high level and multisectoral partnerships contributed to achieving a good health governance system for timely response to the pandemic in Ghana. Also, this strong leadership, efficient management of all resources, and a well-established information system were considered the facilitators for effective mass COVID-19 testing. These findings support studies showing that collective effort from diverse sectors to build a mass testing capacity by increasing the availability of health workers, establishing more testing centers, and reporting consistent data has a positive impact on encouraging more people to get tested and steer its scale-up strategies (8, 27, 28). Taken together, the findings also highlight why and how these factors influence people's behaviors to get tested and public and private actions to cut the COVID-19 transmission.

In contrast, uneven human resources distribution in COVID-19 testing centers, inadequate financial and material resources in a certain context, diffusion of COVID-19 infodemic, and fewer educational opportunities for vulnerable populations were highlighted as the participants complained about barriers against extensive COVID-19 testing. These findings have reminded us of the persistent inequitable health workforce distribution problem, and there is an urgent need to start addressing it now by redistributing existing critical health professions. The findings are consistent with the literature that shortage of supplies/PPE, human resources, and space constraints limit the expansion of COVID-19 testing capacity. Furthermore, scarce resources pose a challenge to ensuring adequate access to testing, especially for vulnerable populations (28). According to Roger et al. (29), the diffusion of misconceptions on COVID-19 that is considered one of the challenges many countries are experiencing is founded on falsehood fabrications around transmission, and infection has generated fearful perceptions about infected people. In addition, fake information about COVID-19 has misled people into low adherence to health protocols (29, 30). The findings on the necessity of education for women in suburban regions support studies addressing consistent community sensitization on major infectious disease risks, including active community engagement, such as providing education for the vulnerable (30, 31).

Even though this study was conducted right before the omicron variant was first reported to WHO, findings from the study would be a foundational basis for developing pandemic preparedness strategies for emerging pandemics. For example, South Korea experienced the largest outbreak, notably the Middle East Respiratory Syndrome (MERS) in 2015, which caused anxiety and confusion in public and significantly impacted the nationwide perception of emergency preparedness. This triggered the reinforcement and strengthening of the country's infectious disease response system. It was extensively restructured together with human resources strengthening with institutional and regulatory change, such as the introduction of special legislation and overarching support from all different sectors (32, 33). It enabled South Korea to successfully control the virus, COVID-19, without lockdowns and business closures. In this context, identified enablers and barriers to COVID-19 testing in Ghana are keys to preparedness, targeting infectious disease prevention. According to the National Institute of Allergy and Infectious Disease(NIH) (34), the pivotal factors of pandemic preparedness strategies are effective communication with the public about prevention practices, aggressive testing and contact tracing, and a strict quarantine policy accompanied by collective support from communities. Those significant factors are clearly drawn up from our findings, and lessons from Ghana may be relevant to all countries with similar contextual settings. This study provides policy implications and insights into how challenges toward COVID-19 testing expansion can be overcome and how multi-and cross-sectoral engagement can strengthen health systems and policies to respond to emerging infections and upscaling testing collaboratively.

### Implications for Policy and Practice

Preliminary policy implications were drawn from data analysis. We anticipate the following recommendations could help stimulate strategies for scaling up COVID-19 testing and developing a pandemic preparedness plan:

- Strong multilateral political commitment is required to ensure adequacy and even distribution of resources to strengthen testing capacity at all levels of health care delivery across Ghana.
- Effective coordination and communication with internal and external partners in Ghana, other countries, international organizations, and private sectors, including biomedical research-oriented philanthropies, are central to ensuring all national pandemic preparedness efforts.
- Empowerment of community members is required to develop and implement community-led pandemic preparedness and management of COVID-19 infodemic to help reduce its impact on negative health behaviors during health emergencies.
- More investment in capacity for process development and Good Manufacturing Practice (GMP), allowing for adaptation when unexpected outbreaks arise, is needed to stimulate the diversification into higher value-added health supplies and products manufactured by local producers in Ghana.
- Incorporating health policy and systems research (HPSR) into the post-COVID-19 pandemic recovery process and future pandemic preparedness is needed to stimulate the engagement of relevant stakeholders to help create stronger health systems responding to emerging pandemics 2.

### Strengths and Limitations of the Study

This study has strengths and limitations. To the best of available literature, COVID-19 testing strategies have not been thoroughly studied yet. This is the first study of its kind to explore facilitators and barriers of COVID-19 testing in Ghana. We were able to explore information from different stakeholders with diverse backgrounds and experiences, thereby enhancing the comprehensiveness and rigor of our findings relevant to decision-making.

Regarding the limitation, the interviews were conducted virtually. This limited us from performing field observation and Focus Group Discussions (FGDs), which we believe could have enhancedstudy rigor. Nevertheless, this does not limit the credibility of our findings as data were compared with existing literature to enhance data triangulation. This study included participants only based in Greater Accra and peripheral of Accra. We, therefore, encourage further studies to be conducted beyond the boundaries of Accra.

## Conclusion

As vaccines are rolled out, testing will continue to play a vital role in controlling COVID-19. The main reason is that testing, followed by contact tracing and isolation of those with positive test results, will promptly allow health professionals to monitor the dynamics of the pandemic. Moreover, according to our findings, COVID-19 testing is still of particular importance to effectively controlling the transmission of the virus in Ghana. Most of the participants confirmed that testing, as an important prevention measure, should be secured with adequate resources and stable health systems. Also, good health governance and leadership, effective resource management, and digitalized information system are successful factors influencing extensive COVID-19 testing. However, upscaling testing capabilities and facilities is faced with several bottlenecks, such as uneven resource distribution, COVID-19 infodemic, and constraints to service delivery. From the analysis, multilateral cooperation and joint partnerships with diverse stakeholders will play a critical role in facilitating active community participation, investment in GMP, and multilateral political commitment in taking bold actions to build strategies to respond to emerging pandemics. Also, a new research area, HPSR, will be a stimulus for many countries to restructure and develop stronger health systems for future pandemics.

## Data Availability Statement

The raw data supporting the conclusions of this article will be made available by the authors, without undue reservation.

## Ethics Statement

The studies involving human participants were reviewed and approved by the Ghana Health Service Ethics Review Committee (No: GHC-ERC 05/07/2021). The patients/participants provided their written informed consent to participate in this study.

## Author Contributions

SH, SY, and AA conceived the study, performed data collection and analysis, and developed the manuscript. The final manuscript was approved by all the authors.

## Conflict of Interest

The authors declare that the research was conducted in the absence of any commercial or financial relationships that could be construed as a potential conflict of interest.

## Publisher's Note

All claims expressed in this article are solely those of the authors and do not necessarily represent those of their affiliated organizations, or those of the publisher, the editors and the reviewers. Any product that may be evaluated in this article, or claim that may be made by its manufacturer, is not guaranteed or endorsed by the publisher.

## Abbreviations

CARES: COVID-19 Alleviation and Revitalization of Enterprises Support
CHPS: Community-Based Health Planning and Services
CNTF: COVID-19 National Trust Fund
EPHF: Essential Public Health Functions
FGDs: Focus Group Discussions
HPSR: Health Policy and Systems Research
MERS: the Middle East Respiratory Syndrome
NCCE: National Commission for Civic Education
NHIS: National Health Insurance Scheme
PHC: Primary Health Care
PPE: Personal Protective Equipment
SDGs: Sustainable Development Goals
SORMAS: Surveillance Outbreak and Response Management and Analysis System
UHC: Universal Health Coverage
WHO: World Health Organization.
